# The pre-conception maternal exposure to Sofosbuvir affects the mitochondrial biogenesis in prenatal fetal tissues: Experimental study on rats

**DOI:** 10.1186/s10020-023-00666-x

**Published:** 2023-06-06

**Authors:** Shimaa A. Mahmoud, Maryam M. Abdel-Aziz, Rana H. M. Khafaga, Hala A. Hafez, Maher A. Kamel, Sara A. Shaker

**Affiliations:** Department of Biochemistry, Medical Research Institute, Alexandria University, 165 El-Horreya Avenue, EL-Hadara, P.O. Box 21561, Alexandria, Egypt

**Keywords:** Hepatitis C virus, Mitochondrial biogenesis, Sofosbuvir, Pregnancy outcome

## Abstract

**Background:**

Hepatitis C virus (HCV) infection is a global public health problem and Egypt has the highest HCV prevalence worldwide. Hence, global efforts target to eliminate HCV by 2030. Sofosbuvir is a nucleotide analogue inhibitor of HCV polymerase essential for viral replication. Animal studies prove that Sofosbuvir metabolites cross the placenta and are excreted in the milk of nursing animals. We aimed to investigate the possible effects of preconception maternal exposure to Sofosbuvir on mitochondrial biogenesis in prenatal fetal liver, skeletal muscle, and placental tissues.

**Methods:**

The study was conducted on 20 female albino rats divided into a control group receiving a placebo and an exposed group receiving 4 mg/kg orally/day for 3 months of Sofosbuvir. At the end of the treatment period, pregnancy was induced in both groups by mating with healthy male rats overnight. At gestational day 17, all pregnant female rats were sacrificed. Each fetus was dissected to obtain the fetal liver, skeletal muscle, and placental tissues.

**Results:**

The results of our study indicated that the exposure of young female rats to Sofosbuvir affects pregnancy outcomes. Fetal liver and muscle showed lower mitochondrial DNA-copy number (mtDNA-CN) by about 24% and 29% respectively, peroxisome proliferator-activated receptor-gamma coactivator-1 alpha and its downstream targets; nuclear respiratory factor-1 and mitochondrial transcription factor A. While the placental tissues showed different patterns, particularly elevated in mtDNA-CN by about 43%.

**Conclusions:**

The study provides preliminary evidence of the detrimental effects of Sofosbuvir on the pregnancy outcomes of the exposed females and may impair the placental and fetal organs’ development. These effects may be mediated through modulating mitochondrial homeostasis and functions.

## Introduction

Hepatitis C virus (HCV) infection is a major public health problem worldwide with an estimated global prevalence of 1% that accounts for more than 71 million infections (Spearman et al. 2019), and a high mortality rate that reaches 400,000 deaths worldwide. Thus, the WHO sets a global target to eliminate HCV as a public health threat by 2030 (Dore and Bajis 2021). Egypt has seroprevalence among the age group 15–59 years about 10% (Omran et al. 2018) which is considered the highest HCV prevalence worldwide, with genotype 4 dominating in more than 90% of all Egyptian patients.

In an attempt to control the situation in Egypt, the Ministry of Health established the National Committee for Control of Viral Hepatitis (NCCVH) which set a national treatment program aiming to decline the prevalence to less than 1% by 2030. In 2014, the National Committee provided Sofosbuvir and other direct-acting antiviral (DAA) agents at a very low cost for patients treated through the national program. Sofosbuvir is a uridine nucleotide analog inhibitor of the viral NS5B polymerase required for viral replication. It interferes with the nascent viral RNA arresting its replication (Gomaa et al. 2017).

Mitochondrial dysfunction has been reported as an off-target effect of most DAA agents (Kim et al. 2017). These agents exert mitochondrial toxicity by inhibiting mitochondrial DNA polymerase γ (pol γ) encoded by the POLG gene, thus halting mitochondrial DNA (mtDNA) replication which leads to mtDNA depletion, increased reactive oxygen species (ROS) production and decreased synthesis of electron transport chain (ETC) proteins (Stoker et al. 2019). Mitochondrial homeostasis is maintained by the coordination between mitochondria biogenesis and mitochondrial degradation (mitophagy). Peroxisome proliferator-activated receptor-gamma coactivator-1 alpha (PGC-1α) controls mitochondrial biogenesis through stimulating the expression of a series of nuclear transcription factors including nuclear respiratory factor-1 (NRF-1) which increases the expression of the mitochondrial transcription factor A (Tfam), the final effector of mtDNA transcription and replication (Popov 2020). Regarding mitochondrial biogenesis, the antiretroviral drugs have an impact on mtDNA copy number (mtDNA-CN) and the main regulators of mitochondrial biogenesis, PGC-1α, and NRF-1. These mitochondrial effects may interfere with and impact the functions of the tissues depending on the type of the affected tissues and also depending on the developmental age (Viengchareun et al. 2007).

When facing bioenergetic or oxidative challenges, mitochondria exhibit a graded response that involves changes in their morphology and dynamics through the activation of the distinct molecular mechanisms that regulate mitochondrial fission, fusion, mitophagy, and mitochondrial biogenesis. Mitochondrial fission and fusion are tightly regulated by complex protein machinery involving dynamin-related protein 1(DRP1), mitofusin 1 (MFN1), mitofusin 2 (MFN2), and Optic atrophy protein 1 (OPA1) in mammalian cells. Mitochondrial fission was found to contribute to mitochondrial apoptosis and was also suggested to be a prerequisite for mitophagy, while mitochondrial fusion is linked to an increase in mitochondrial metabolism. It is important to address these questions, as mitochondrial dynamics and homeostasis are tightly linked with cellular physiology and eventually cell fate (Ma et al. 2020).

The strategy of the National Committee for Control of Viral Hepatitis (NCCVH) to control HCV infection in Egypt was to treat every infected individual with DAA, especially Sofosbuvir. This mass treatment strategy during the last years involved many young, infected females of childbearing age which attract our attention to the possible long-lasting adverse effects of Sofosbuvir on the pregnancy and offspring of exposed mothers (Waked et al. 2020). Our previous work indicated disturbed mitochondrial biogenesis and functions in different tissues of young female rats exposed to the therapeutic dose of Sofosbuvir for three months. The ovarian tissue appears to be the most affected organ followed by the hepatic tissue and the skeletal muscles which means that Sofosbuvir exposure not only affects the health of the exposed females but also affects the future offspring of these females (unpublished data).

During pregnancy, the in-utero environment is the vital element that programs to a large extent the health of an individual throughout life because the mammalian fetus is completely dependent on the nutrients supplied by its mother, and any change in this supply or shift in maternal metabolic status can alter fetal development structurally and functionally. This effect has been described as the “fetal origin of adult disease” (Kamel 2012). So, we designed this study to examine the possible effects of the pre-conception exposure of young female rats to Sofosbuvir on the pregnancy outcome and the mitochondrial biogenesis and function in different fetal tissues and placental tissue prenatally.

## Materials and methods

### Experimental animals

The study was conducted on 20 female Wistar albino rats about 2 months old weighing 115–125 g. Rats were obtained from the animal house facility in Medical Technology Center, Medical Research Institute, Alexandria University, Egypt. Animals were kept 5 per cage at 23 °C in a 12 h light/12 h dark cycle under good hygienic conditions and standard humidity with access to food and water.

### Ethical statement

The study was approved by the Institutional Animal Care and Use Committee (IACUC)-Alexandria University, Egypt in 2020 (AU0122012113). All steps were performed following the guidelines for the care and use of laboratory animals (USA National Institute of Health Publication No 80–23, revised 1996) and all efforts were made to reduce the distress of rats during the whole experimental period.

### Drug

Sofosbuvir was available in the form of tablets with the trade name ‘Gratisovir’ (a product of Pharco Pharmaceuticals, Alexandria, Egypt), Each tablet contains 400 mg of Sofosbuvir; these tablets were dissolved in distilled water and given to rats orally by gastric tube in a dose of 4 mg/kg/day for 3 months (Issa and El-sherief 2017; Abdeen et al. 2019).

### Experimental design

The animals were classified into two groups according to the treatment they were receiving:

- **Group I** (Control group): 10 healthy female rats that were maintained under a normal diet and received a placebo for 3 months.
- **Group II** (exposure group): 10 healthy female rats that were supplemented with 4 mg/kg of Sofosbuvir orally by gastric tube once per day for 3 months (Issa and El-sherief 2017; Abdeen et al. 2019).

#### Induction of pregnancy

- After the three months of treatments, the pregnancy was induced in control and Sofosbuvir-exposed females by mating with healthy male rats overnight in a ratio (2 females: 1 male). The next day was considered day 0 of pregnancy (gestational Day 0).
- At gestational day 17 (GD 17), 10 pregnant females of each group were sacrificed by cervical dislocation under deep anesthesia with ketamine 100 mg/kg and xylazine 10 mg/kg.

#### Collection of samples

Fetuses with their membranes and placentas were quickly dissected out of the uterine horns. The number of resorbed (dead) fetuses for each pregnancy was noted and excluded from the study. Each fetus and its membrane were separated by gentle dissection and rinsed carefully in phosphate buffer saline (PBS). Overall growth and differentiation of the fetus were quantified by direct measurement of crown-rump length. The weights of fetuses and placentas were recorded. The liver, muscle, and placental tissues of 10 fetuses were obtained, washed, and each organ was divided into 3 aliquots; the first one was used for DNA isolation for the assessment of mitochondria DNA copy number (mtDNA-CN), the second was used for total RNA isolation for the assessment of genes expression, and the third aliquot was homogenized in phosphate-buffered saline (0.1 M, pH 7.4) in ratio1:9 and centrifuged at 10,000 × *g* for 10 min at 4 °C and the supernatant was stored in aliquots for subsequent determinations of total protein level by Lowry method, malondialdehyde (MDA), Glutathione and the protein levels of NADH dehydrogenase subunit-5 (ND5), PGC-1α, Tfam, NRF-1, and Nuclear factor-kappa B (NF-κB) by ELISA and citrate synthase (CS) activity.

### Tissues contents of NADH dehydrogenase subunit-5 using ELISA

The supernatant was used for NADH dehydrogenase subunit-5 (ND5) determination using ELISA assay (Chongqing Biospes Co., China) according to the manufacturer’s instructions. The total protein concentration was determined using Lowry’s method (Lowry et al. 1951).

### Tissue's activities of citrate synthase

Citrate synthase activity can be measured through the formation of the -SH group released from CoA-SH by use of the reactive Ellman reagent (5,5`-dithiobis [2-nitrobenzoic], DTNB) and monitoring the absorbance at 412 nm (Shepherd and Garland 1969).

### Tissues contents of total reduced and oxidized glutathione

Glutathione (GSH) and glutathione disulfide (GSSG) were assayed using the method of Griffith which depends on the oxidation of GSH by 5,5`-dithiobis-(2-nitrobenzoic acid) (DTNB) to yield GSSG and 5-thio-2-nitrobenzoic acid (TNB). Oxidized GSSG is reduced enzymatically by the action of glutathione reductase and NADPH to regenerate GSH. The rate of TNB formation is monitored at 412 nm and is proportional to the sum of GSH and GSSG present in the sample (Griffith 1980).

### Determination of malondialdehyde (MDA) as thiobarbituric acid reactive substances (TBARS)

Malondialdehyde was determined according to the method of Draper and Hadley. The tissue samples are heated with thiobarbituric acid (TBA) at low pH. The resulting pink chromogen has a maximal absorbance at 532 nm (Draper and Hadley 1990).

### Tissues mRNA expression of PGC-1α, Tfam, NRF-1, NF-KB, POLG, DRP1, MFN1 and OPA1

Thirty mg of the liver, muscle, or placental tissues were used for total RNA extraction using the miRNeasy Mini Kit (Qiagen, Germany) according to the manufacturer’s instructions and the concentration and integrity of extracted RNA were checked using nanodrop. The reverse transcription of the extracted RNA was performed using Reverse transcription (RT) performed by TOPscript™ RT DryMIX kit (dT18/dN6 plus) (Enzynomics, Korea) according to the manufacturer's instructions. The tissues expression of PGC-1α, Tfam, NRF-1, nuclear factor kappa B (NF-κB), and DNA polymerase gamma (POLG) POLG, DRP1, MFN1 and OPA1 were quantified in the cDNA by CFX Maestro™ Software (Bio-Rad, USA) using QuantiNova™ SYBR® Green PCR Kit (Qiagen, Germany). Quantitative PCR amplification conditions were adjusted as an initial denaturation at 95 °C for 10 min and then 45 cycles of PCR for amplification as follows: Denaturation at 95 °C for 20 s, annealing at 55 °C for 20 s and extension at 70 °C for 15 s. The housekeeping gene 18S rRNA was used as a reference gene for normalization. The primers used for the determination of rat genes are presented in Table 1. The relative change in mRNA expression in samples was estimated using the 2-^ΔΔCt^ method.

**Table 1:** Primers used for the PCR amplification

Gene	Accession number	primer sequence	
18S rRNA (Reference gene)	NR_046237.2	F:	GTAACCCGTTGAACCCCATT
		R:	CAAGCTTATGACCCGCACTT
PGC-1α	NM_031347.1	F:	GTGCAGCCAAGACTCTGTATGG
		R:	GTCCAGGTCATTCACATCAAGTTC
NRF-1	NM_001100708.1	F:	TTACTCTGCTGTGGCTGATGG
		R:	CCTCTGATGCTTGCGTCGTCT
Tfam	NM_031326.2	F:	CCCACAGAGAACAGAAACAG
		R:	CCCTGGAAGCTTTCAGATACG
POLG	NM_053528.1	F:	GGACCTCCCTTAGAGAGGGA
		R:	AGCATGCCAGCCAGAGTCACT
mtDNA	NC_040919.1	F:	AATGGTTCGTTTGTTCAACGATT
		R:	AGAAACCGACCTGGATTGCTC
n-PGC-1α	NM_031347.1	F:	ATGAATGCAGCGGTCTTAGC
		R:	AACAATGGCAGGGTTTGTTC
DRP1	NM_053655.3	F:	GATGCCATAGTTGAAGTGGTGAC
		R:	CCACAAGCATCAGCAAAGTCTGG
OPA1	NM_133585.3	F:	CAGCTGGCAGAAGATCTCAAG
		R:	CATGAGCAGGATTTTGACACC
MFN1	NM_130894.4	F:	GCCAGCTTCCTTGAAGACAC
		R:	GCAGAACTTTGTCCCAGAGC
NF-κB (P65)	NM_199267.2	F:	CAGGACCAGGAACAGTTCGAA
		R:	CCAGGTTCTGGAAGCTATGGAT

Where F: Forward primer and R: Reverse primer

### Tissues mitochondrial DNA copy number (mtDNA-CN)

In the present study, we used a qPCR assay to estimate the abundance of mtDNA relative to nuclear DNA. After total genomic DNA isolation, we used a specific primer pair for mtDNA sequence (mtDNA) and a primer pair specific for nuclear sequence (PGC-1α) to perform the same number of PCR cycles and calculate the relative mtDNA signal to nuclear DNA signal. The nuclear gene was used to quantify nuclear DNA (nDNA) and therefore normalization of the mtDNA amount per the nDNA of the diploid cells using the equation:

$$\begin{gathered} {\mathbf{R}} \, = \, {\mathbf{2}}^{{ - {\mathbf{\Delta Ct}}}}\,{\text{where}}\, {\mathbf{\Delta Ct}} = \, {\mathbf{Ct}}_{{{\mathbf{mtDNA}}}} {-} \, {\mathbf{Ct}}_{{{\mathbf{nuclear}}}}\end{gathered}$$

In this study, total DNA was isolated from the different tissues using a DNeasy kit (Qiagen, USA) according to the manufacturer's instructions. A specific primer pair for mtDNA and a primer pair for the nuclear PGC-1α gene (Table 1) were used. PCR reactions were carried out using SYBR Green PCR Master Mix (Qiagen, Germany), 0.5 μM of each primer pair, and 50 ng genomic DNA under the following conditions: 95˚C for 10 min followed by 40 cycles of 95 °C for 15 s, 60 °C for 30 s and 72 °C for 30 s (Gowayed et al. 2020).

### Tissues protein levels of PGC-1α, Tfam, NRF-1, and NF-κB by ELISA

The tissue contents of PGC-1α, Tfam and NRF1 were assayed using specific rat ELISA kits (MyBioSource, San Diego, USA) according to the instructions of the manufacturer. Also, the tissue NF-κB was assayed using specific rat ELISA kits obtained from CUSABIO Co. (Houston, USA).

### Statistical analysis

Data were analyzed using SPSS software package version 18.0 (SPSS, Chicago, IL, USA). The Kolmogorov–Smirnov test was used to study the normal distribution of the studied parameters. The data were expressed as mean ± SD and analyzed using the Student’s (independent) t-test to compare different groups and Pearson for correlation studies. The p-value was assumed to be significant at p < 0.05 (Hagen 2002).

## Results

### Pregnancy outcome

The pregnancy outcome of control and Sofosbuvir-exposed mothers is illustrated in Table 2. Sofosbuvir-exposed mothers showed decreased number of viable fetuses per litter by about 29% compared with control pregnancies. Moreover, the pregnancies of Sofosbuvir-exposed mothers have a marked increase in the resorbed (dead) fetuses by about 375% compared with the pregnancies of control mothers.

**Table 2:** Pregnancy outcome of control and Sofosbuvir-exposed mothers

Groups	Number of resorbed fetus/litter	Number of viable fetus/litter	Weight (g)		Fetal Crown-rump length (cm)
			Fetus	Fetal placenta	
Control	0.8 ± 0.42	13.2 ± 3.1	0.77 ± 0.09	0.38 ± 0.06	1.7 ± 0.14
Sofosbuvir exposed	3.8^*^ ± 1.7	9.4 ± 2.9	1.68^*^ ± 0.36	0.47^*^ ± 0.08	2.33^*^ ± 0.24
% of change from control	375	− 28.8	118.1	35.13	37

Data were illustrated as Mean ± SD

Number of fetuses in each group = 10

*g* gram

*cm* centimeter

*p < 0.05, indicating a statistically significant difference when compared with the control group using independent sample t-test

The fetal placentas of Sofosbuvir-exposed mothers were significantly heavier than the placentas of the fetuses of control mothers by about 35%. The fetal growth was assayed by fetal weight and crown-rump length (CRL). The results indicated that the fetuses of Sofosbuvir-exposed mothers are significant heavier (increased weight) and longer (long CRL) than about (CRL) by about 118% and 37%, respectively, compared with the fetuses of control mothers.

### Malondialdehyde (MDA) content in different tissues

The content of MDA was significantly increased in all tissues of fetuses of Sofosbuvir-exposed mothers by about 74% in the liver, 144% in muscle and 667% in the placenta compared with fetuses of control mothers (Table 3).

**Table 3:** The change of Malondialdehyde (MDA) content in the studied fetal tissues

Groups	MDA (nmol/mg protein)		
	Fetal liver	Fetal muscle	Fetal placenta
Control	0.78 ± 0.039	1.7 ± 0.32	0.30 ± 0.034
Sofosbuvir exposed	1.36^*^ ± 0.15	4.15^*^ ± 0.22	2.33* ± 0.39
% of change from control	74.3	144.1	667.6

Data were illustrated as Mean ± SD

Number of fetuses in each group = 10

MDA Malondialdehyde

*p < 0.05, indicating a statistically significant difference when compared with the control group using independent sample t-test

### Glutathione contents in different tissues

**Fig. 1:**
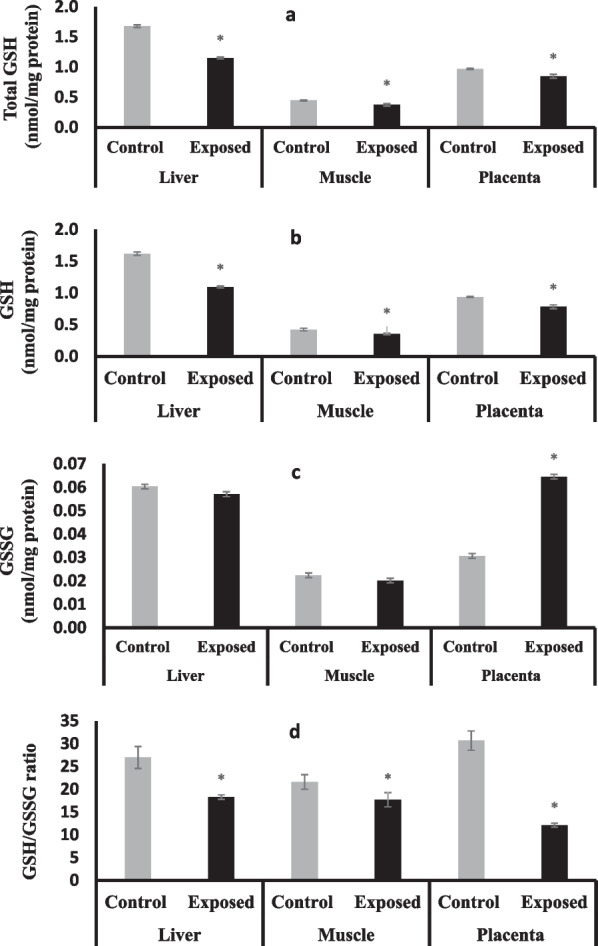
Total (**a**), reduced (**b**) oxidized (**c**) glutathione, GSH/GSSG ratio (**d**) in the fetal tissues**.** Data were illustrated as Mean ± SD**,** Number of fetuses in each group = 10**,** ^*^p < 0.05, indicating a statistically significant difference when compared with control group using independent sample t-test

Fetuses of Sofosbuvir-exposed mothers showed a significant decrease in total glutathione (t GSH) and reduced glutathione (GSH) in all tissues compared with fetuses of control mothers (Fig. 1a, b) especially in the fetal liver.

Fetuses of Sofosbuvir-exposed mothers showed a significant increase in oxidized glutathione (GSSG) in placental tissue however showed no significant changes were observed in liver and muscle tissues compared with fetuses of control mothers **(**Fig. 1c**).**

Fetuses of Sofosbuvir-exposed mothers showed a significant decrease in GSH/GSSG in all tissues compared with fetuses of control mothers, especially in placental tissues **(**Fig. 1d**).**

### NADH dehydrogenase subunit-5 (ND5) content in different tissues

**Fig. 2:**
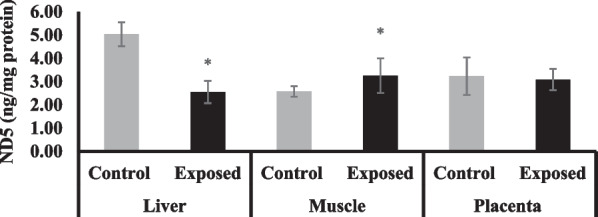
The change of NADH dehydrogenase subunit-5 content in the fetal tissues. Data were illustrated as Mean ± SD**,** Number of fetuses in each group = 10**,** ^*^p < 0.05, indicating a statistically significant difference when compared with control group using independent sample t-test

The content of ND5 was significantly lower by about 50% in the liver of fetuses of Sofosbuvir-exposed mothers compared with fetuses of control mothers, while fetal muscle has significantly higher ND5 content than the fetuses of control mothers by about 53%. The placental tissues of fetuses of Sofosbuvir-exposed mothers show a non-significant decrease in ND5 content by about 17% compared with the control placentas (Fig. 2).

### Citrate synthase (CS) activity in different tissues

**Fig. 3:**
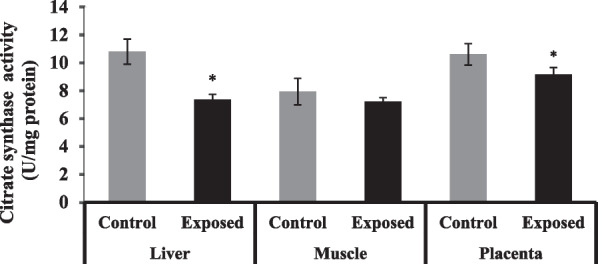
The change of CS activities in the fetal tissues. Data were illustrated as Mean ± SD**,** Number of fetuses in each group = 10**,** ^*^p < 0.05, indicating a statistically significant difference when compared with control group using independent sample t-test

The activities of CS were significantly lower by about 90% in the liver and placenta of fetuses of Sofosbuvir-exposed mothers compared with fetuses of control mothers, while fetal muscle has no significant difference in CS activity compared between the two groups (Fig. 3).

### Mitochondrial DNA copy number (mtDNA-CN) in different tissues

**Fig. 4:**
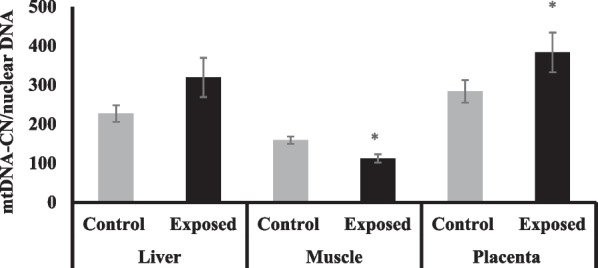
The change of mtDNA-CN/nuclear DNA in the fetal tissues. Data were illustrated as Mean ± SD**,** Number of fetuses in each group = 10**,** ^*^p < 0.05, indicating a statistically significant difference when compared with control group using independent sample t-test

The fetal placentas of Sofosbuvir-exposed mothers have significantly higher mtDNA-CN by about 43% compared with fetuses of control mothers. The fetal livers show a mild but non-significant increase by about 24% compared with fetuses of control mothers. In contrast, the fetal muscle of fetuses of Sofosbuvir-exposed mothers has significantly lower mtDNA-CN compared with the fetuses of control mothers by about 29% (Fig. 4).

### Peroxisome proliferator-activated receptor-gamma coactivator-1 alpha (PGC-1α)

**Fig. 5:**
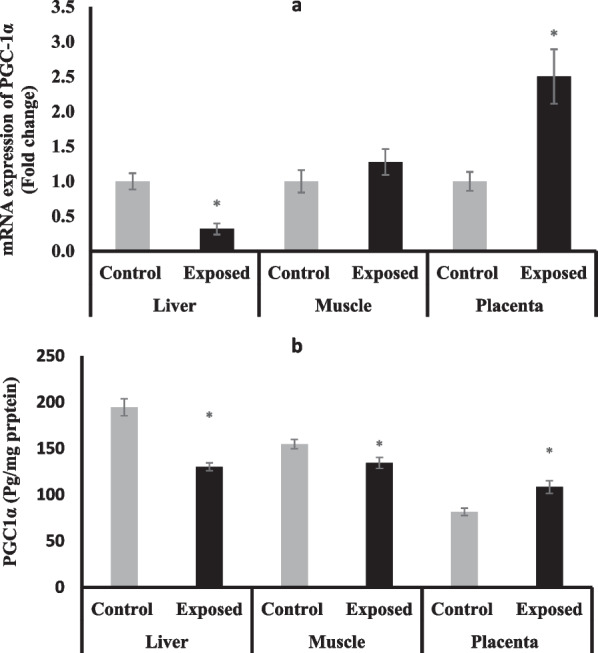
The change of PGC-1α expression at mRNA (**a**) protein (**b**) levels in the fetal tissues. Data were illustrated as Mean ± SD**,** Number of fetuses in each group = 10**,** ^*^p < 0.05, indicating a statistically significant difference when compared with control group using independent sample t-test

At the mRNA level, the expression of PGC-1α was significantly induced in the placental tissues of fetuses of Sofosbuvir-exposed mothers to be 2.5-fold compared with fetuses of control mothers. In contrast, the expression of PGC-1α was significantly suppressed in the liver tissues of fetuses of Sofosbuvir-exposed mothers by about 68% compared with fetuses of control mothers. The expression of PGC-1α showed a mild but non-significant increase by about 28% in the muscle of fetuses of Sofosbuvir-exposed mothers compared with fetuses of control mothers (Fig. 5a).

At the protein level, the liver and muscle tissues of fetuses of Sofosbuvir-exposed mothers have significantly lower PGC-1α protein levels compared with the control fetuses. In contrast, the placental tissues of the exposed mothers showed significantly higher protein levels compared with the control **(**Fig. 5b**).**

### Nuclear respiratory factor-1 (NRF-1)

**Fig. 6:**
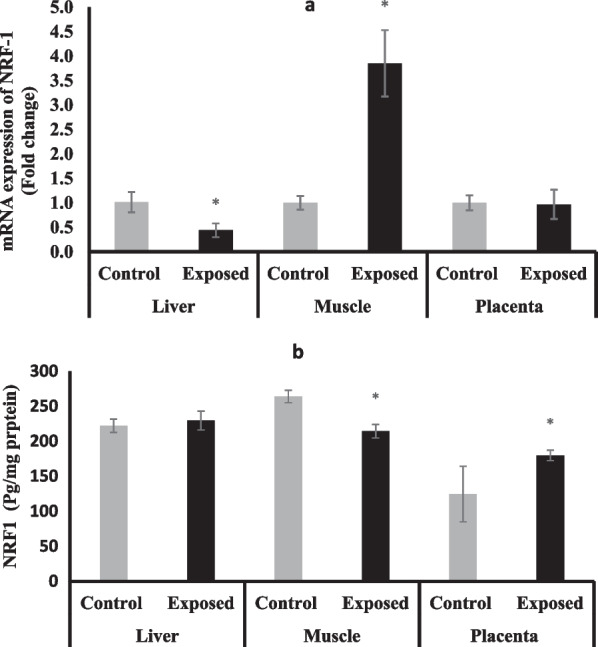
The change of NRF-1 expression at mRNA (**a**) protein (**b**) levels in the fetal tissues. Data were illustrated as Mean ± SD**,** Number of fetuses in each group = 10**,** ^*^p < 0.05, indicating a statistically significant difference when compared with control group using independent sample t-test

The expression of NRF-1 at mRNA level was significantly suppressed in liver tissues of fetuses of Sofosbuvir-exposed mothers compared with fetuses of control mothers by about 56%. In contrast, NRF-1 expression was significantly upregulated in the skeletal muscle tissues of fetuses of Sofosbuvir-exposed mothers to be about fourfold the control value. The placental tissues show no significant change in the expression of NRF-1 in the fetuses of Sofosbuvir-exposed mothers compared with the fetuses of control mothers (Fig. 6a).

The level of NRF-1 protein was significantly lower in the fetal muscle of the fetuses of exposed mothers compared with the control fetuses, while it was significantly higher in the placental tissues. No significant changes were observed in the NRF-1 protein in the fetal liver **(**Fig. 6b**).**

### Mitochondrial transcription factors-A (Tfam)

**Fig. 7:**
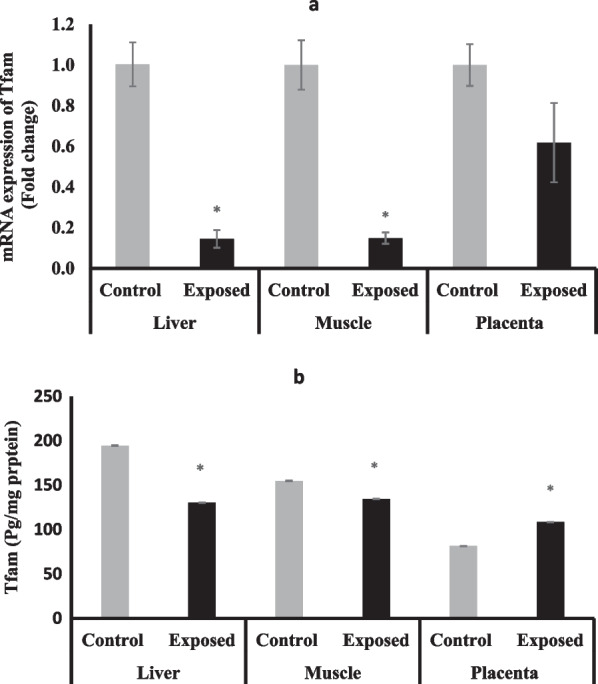
The change of Tfam expression at mRNA (**a**) protein (**b**) levels in the fetal tissues. Data were illustrated as Mean ± SD**,** Number of fetuses in each group = 10**,** ^*^p < 0.05, indicating a statistically significant difference when compared with control group using independent sample t-test

The expression of Tfam at mRNA level was significantly suppressed in the liver and skeletal muscle of fetuses of Sofosbuvir-exposed mothers by about 85% compared with fetuses of control mothers, while the placental tissues of fetuses of Sofosbuvir-exposed mothers have lower but not significant Tfam expression compared with the control placentas (Fig. 7a**)**.

The protein levels of Tfam showed significantly lower levels in the fetal liver and muscles however, significantly higher in the placentas of exposed mothers compared with the control fetuses (Fig. 7b).

#### Dynamin-related protein 1 (DRP1) gene expression in different tissues

**Fig. 8:**
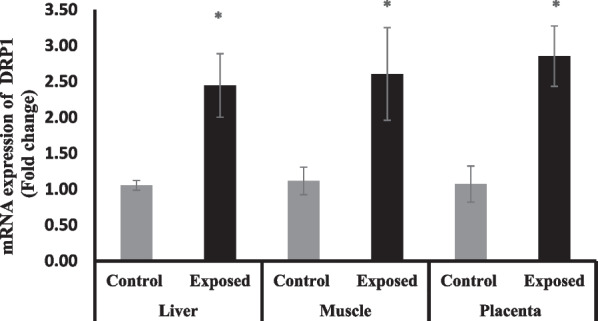
The change of DRP1 gene expression in the fetal tissues. Data were illustrated as Mean ± SD**,** Number of fetuses in each group = 10**,** ^*^p < 0.05, indicating a statistically significant difference when compared with control group using independent sample t-test

The expression of DRP1 was significantly induced in the liver, skeletal muscle and placental tissues of fetuses of Sofosbuvir-exposed mothers compared with fetuses of control mothers by about 131%, 133%, and 167% respectively (Fig. 8).

#### Optic atrophy protein 1 (OPA1) expression in different tissues

**Fig. 9:**
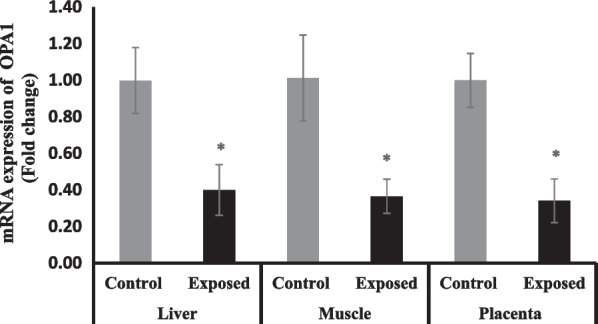
The change of OPA1 gene expression in the fetal tissues. Data were illustrated as Mean ± SD**,** Number of fetuses in each group = 10**,** ^*^p < 0.05, indicating a statistically significant difference when compared with control group using independent sample t-test

The expression of OPA1 was significantly suppressed in the liver, skeletal muscle and placental tissues of fetuses of Sofosbuvir-exposed mothers compared with fetuses of control mothers by about 60% (Fig. 9).

#### Mitofusin (MFN1) expression in different tissues

**Fig. 10:**
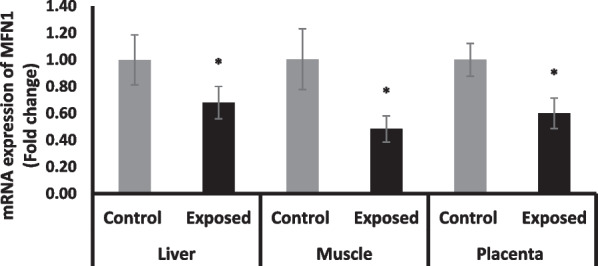
The change of MFN gene expression in the fetal tissues. Data were illustrated as Mean ± SD**,** Number of fetuses in each group = 10**,** ^*^p < 0.05, indicating a statistically significant difference when compared with control group using independent sample t-test

The expression of MFN1 was significantly declined in the liver, skeletal muscle and placental tissues of fetuses of Sofosbuvir-exposed mothers compared with fetuses of control mothers by about—32%, 52%, 40%. respectively (Fig. 10).

### Nuclear factor-kappa B expression (NF-κB)

**Fig. 11:**
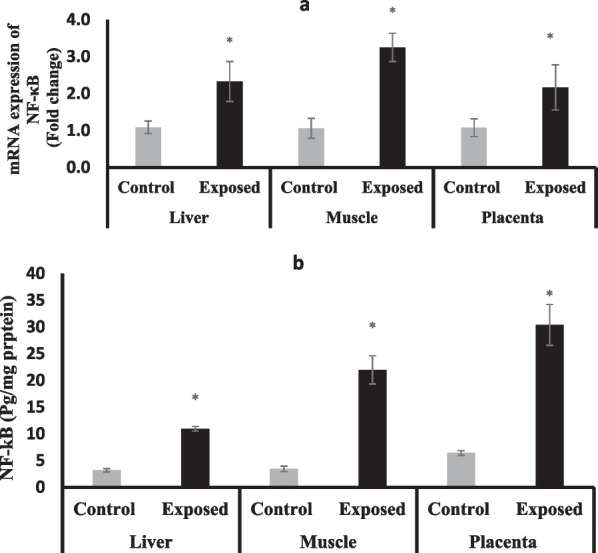
The change of NF-κB expression at mRNA (**a**) protein (**b**) levels in the fetal tissues. Data were illustrated as Mean ± SD**,** Number of fetuses in each group = 10**,** ^*^p < 0.05, indicating a statistically significant difference when compared with control group using independent sample t-test

The expression of NF-κB at mRNA level was significantly induced in all tissues of fetuses of Sofosbuvir-exposed mothers to be 2.5-fold in the liver, threefold in muscle, twofold in the placenta compared with fetuses of control mothers (Fig. 11a). Also, at the protein level, the NF-κB showed significantly higher levels in the three studied tissues, especially in the placentas and muscles of the fetuses of the exposed mothers (Fig. 11b).

### Tissue expression of mitochondrial DNA polymerase gamma (POLG)

**Fig. 12:**
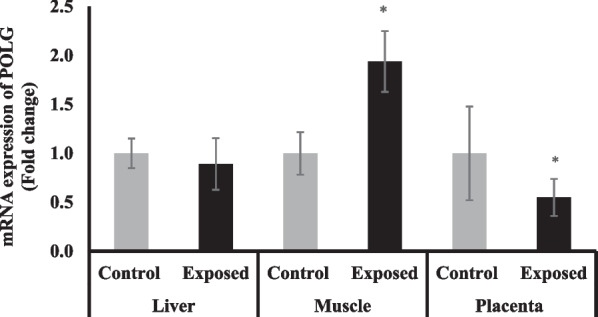
The change of POLG gene expression in the fetal tissues. Data were illustrated as Mean ± SD**,** Number of fetuses in each group = 10**,** ^*^p < 0.05, indicating a statistically significant difference when compared with control group using independent sample t-test

The expression of POLG was significantly suppressed in placental tissues of fetuses of Sofosbuvir-exposed mothers by about 45% compared with fetuses of control mothers. In contrast, the expression of POLG was significantly upregulated in skeletal muscle tissues of fetuses of Sofosbuvir-exposed mothers by about 94% compared with the fetuses of control mothers. The liver tissues of fetuses of Sofosbuvir-exposed mothers show a non-significant decrease in the expression of POLG compared with the fetuses of control mothers (Fig. 12).

### Correlation studies

**Fig. 13:**
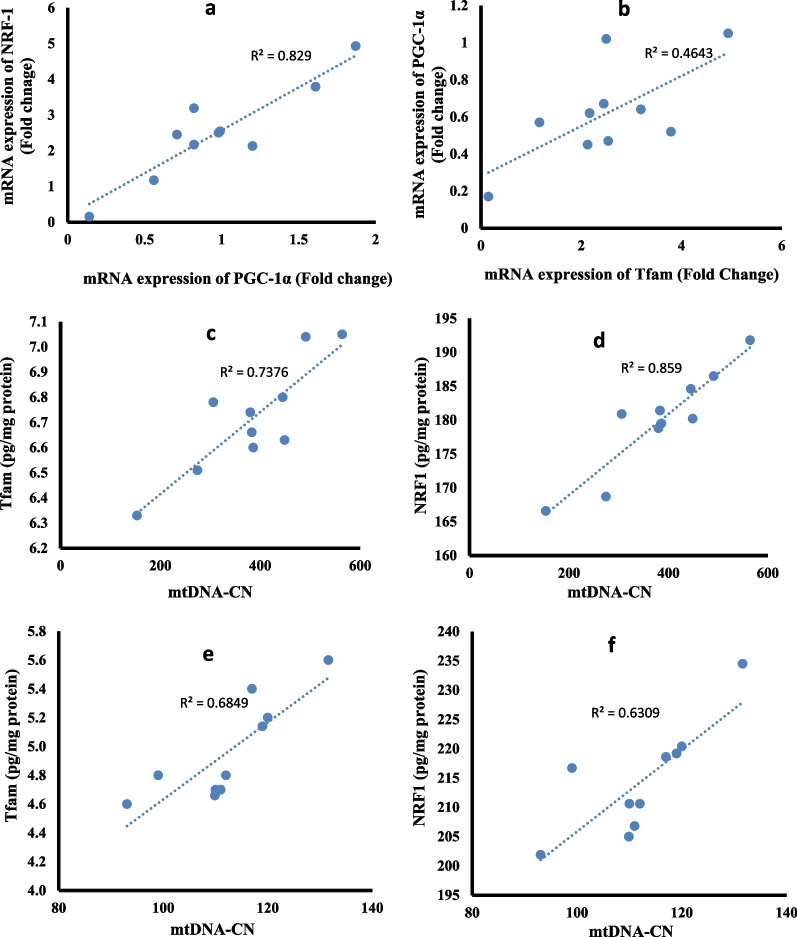
Correlation curves between different parameters in the fetal tissues. (**a**–**d**) correlation curves in placental tissues between PGC-1α expression with NRF-1 expression (**a**) and Tfam expression (**b**), mtDNA copy number with Tfam content (**c**) and NRF1 content (**d**), (**e**–**f**) correlation curves in muscle tissues between mtDNA copy number with Tfam content (**e**) and NRF1 content (**f**)

The statistical analysis using Pearson correlation reveals that PGC-1α expression was positively correlated with NRF-1 expression (r = 0.911, p˂0.001, Fig. 13a) and Tfam expression (r = 0.681, p = 0.030, Fig. 13b) in placental tissue of fetuses of Sofosbuvir-exposed mothers. Also, in placental tissues of the fetuses of exposed mothers, the mtDNA copy number is positively correlated with the protein contents of Tfam (r = 0.86, p = 0.002, Fig. 13c) and NRF1 (r = 0.93, p = 0.001, Fig. 13d). In the muscle tissues of fetuses of exposed mothers, the mtDNA copy number is positively correlated with the protein contents of Tfam (r = 0.83, p = 0.005, Fig. 13e) and NRF1 (r = 0.80, p = 0.01, Fig. 13f).

## Discussion

The results of the present study provide preliminary evidence that the preconception exposure of young female rats to Sofosbuvir has detrimental effects on the pregnancy outcomes of these females and may impair the placental and fetal organs’ development. These effects may be mediated through modulating mitochondrial biogenesis. So, the results raise serious concerns about the safety of sofosbuvir treatment in young females.

In our study, Preconception exposure to Sofosbuvir disrupted normal cellular homeostasis and cause oxidative stress in different fetal tissues (placenta, muscle, and liver) and led to a state of inflammation that was associated with a significant increase in lipid peroxidation marker (MDA), and oxidized glutathione (GSSG) and a significant drop in total and reduced GSH. These changes result in shifting the redox status in the fetal tissues from the reducing environment (high GSH/GSSG ratio) into a more oxidizing environment (low GSH/GSSG ratio). High concentrations of GSH are associated with a reducing environment and increased proliferation, while cell death is initiated by an oxidizing environment (low GSH and GSH/GSSG) (El-Bassiouni et al. 2005). On the other hand, increased mitochondrial ROS in fetal tissues may cause direct damage to mtDNA, thus, disrupting mitochondrial homeostasis. However, in an attempt to maintain normal fetal growth, either increased or decreased mitochondrial biogenesis could take place (Holland et al.^ 2017^).

Preconception exposure to Sofosbuvir causes a substantial decrease in pregnancy outcomes. This effect may be related to the decreased quality of the oocytes due to the three months of exposure to Sofosbuvir which may impair the mitochondrial biogenesis and functions in the ovarian tissues of the mothers before the pregnancy which ultimately results in decreased fertility. This assumption is confirmed by our previous work indicating disturbed mitochondrial biogenesis and functions in the ovarian tissue due to treatment with Sofosbuvir (unpublished data). The low ovarian mtDNA-CN is a marker of poor oocyte quality, which may lead to poor embryonic development (May-Panloup et al. 2016). The maternal mitochondrial dysfunctions and impaired biogenesis may be transmitted to following generations and may cause many diseases (Chiaratti et al. 2018). Also, the possible direct effects of the Sofosbuvir administered before pregnancy cannot be ruled out since animal studies prove that Sofosbuvir metabolites cross the placenta and are excreted in the milk of nursing animals (Spera et al. 2016).

The placental defects may be another contributing factor to the decline in pregnancy outcomes and fetal abnormalities. The placenta is the first organ formed in pregnancy and it connects the maternal and embryonic circulations to facilitate the fetal supply with maternal nutrients (Herrera and Desoye 2016). The placentomegaly observed in the pregnancies of Sofosbuvir-exposed mothers may indicate a state of chronic inflammation which was confirmed in our study by induced placental expression of the pro-inflammatory marker; NF-κB at both mRNA and protein contents. The placental inflammation may compromise its function as a selective barrier for the transport of nutrients and others into the fetus. Thus, the redox status of the placental tissues of Sofosbuvir-exposed mothers indicates a state of cell death and inflammation. The structural changes and maladaptation in the placenta may cause inflammatory shock to the fetus that may result in long-term adverse outcomes, including asthma, cerebral palsy, abnormal neurodevelopment, and autism spectrum disorder (Goldstein et al. 2020^).^

The observed changes in fetal placental size and weight due to maternal exposure to Sofosbuvir are associated with significant changes in mitochondrial homeostasis (fission, fusion, and biogenesis) and functions in fetal tissues and placentas and significant changes in tissues expression of genes controlling mitochondrial biogenesis, mitophagy and functions. However, the patterns of changes are not similar between the different tissues and in the same tissue between the mRNA and protein levels of the same gene.

Regarding the mitochondrial DNA copy number (mtDNA-CN), an indicator of mitochondrial biogenesis, the placental tissues of Sofosbuvir-exposed mothers have significantly higher levels, the fetal liver showed no significant change, however, the fetal muscle has significantly lower levels. The differential effects of the pre-conception maternal exposure to Sofosbuvir on the placenta and fetal tissues may be explained by the fact that the tissues are not homogeneous regarding the mtDNA-CN depending on the energy demand and the metabolic requirements. This explanation agrees with Pejznochova and colleagues who assumed that the non-homogenous distribution of mtDNA between fetal tissues is attributed to the differences in their metabolic roles and that mtDNA-CN in fetal tissues is considered tissue-specific and depends on energy requirements as well as the stage of development (Pejznochova et al. 2010). This could explain the variation in mtDNA-CN in different sampled tissues in our study.

The disturbed mtDNA-CN was associated with significant changes in the expression of genes controlling the pathway of mitochondrial biogenesis. In the placenta, the expression of PGC-α, NRF1, and Tfam showed significant upregulation at the protein level while at the mRNA level only PGC-α showed significant upregulation while NRF1 and Tfam showed no significant changes. The protein levels are the main determinant at the functional levels as indicated by the significant positive correlation between the placenta mtDNA-CN with the protein levels of NRF1 and Tfam. The placental inflammation and oxidative stress may lead to mitochondrial proliferation to compensate for the disrupted cellular bioenergetics.

Besides its key role as the main regulator of mitochondrial biogenesis, PGC-1α has been recognized as a powerful regulator of angiogenesis which is fundamental to the development of a healthy placenta in normal pregnancy. It may be possible that PGC-1α has a key role in the imbalance of angiogenic factors observed in pre-eclampsia. Delany and colleagues suggested that PGC-1α may be implicated in the pathogenesis of pre-eclampsia (Delany et al. 2013). Vishnyakova et al. found increased placental mitochondrial content in early-onset but not late-onset pre-eclampsia, suggesting that the different pathophysiology leads to differences in mitochondrial response (Vishnyakova et al. 2016). The maintenance of mitochondrial biogenesis during the critical period of fetal development is very crucial because the increase in mitochondrial biogenesis during pregnancy is required to maintain the metabolic activity of the placenta. Disruption of this process could increase the production of ROS and oxidative stress which subsequently leads to placental insufficiency and damage that is maybe manifested as pre-eclampsia or intrauterine growth restriction (Saad et al. 2016; Mandò et al. 2014).

Mitochondrial fusion and fission, which are strongly related to normal mitochondrial function, are referred to as mitochondrial dynamics. Regarding DRP1, OPA1 and MFN1, indicators of the mitochondrial dynamics, Sofosbuvir-exposed mothers have significantly lower OPA1 and MFN1 and higher DRP1 expressions in the placenta, fetal liver and fetal muscle. Mitochondrial fusion has a central role in cellular bioenergetics. Typically, it is stimulated in conditions of increased oxidative phosphorylation needs and ‘cellular starvation’, to preserve mtDNA levels, mitochondrial membrane potential and respiratory function. Tissue sensitivity to deficiencies in mitochondrial fusion capacity varies. In embryonic and placental development, for instance, mitochondrial fusion is essential as MFN1 and Mfn2 knock-out in mice results in fetal death due to placental insufficiency. In addition, fusion is one of the first-line mechanisms to repair mitochondrial damage by permitting sharing of content such as mtDNA and lipids (Abbade et al. 2020).

The increased placental mtDNA-CN does not associate with a significant increase in the NADH dehydrogenase subunit-5 (ND5) in placental tissue which even showed a non-significant decline. NADH dehydrogenase, complex I, is the first and largest of the ETC complexes that have a pivotal role in energy metabolism as it is the main entry point for electrons to the ETC, hence, considered the rate-limiting step in overall respiration. Mutations in the genes encoding the complex I subunits have been associated with ETC disturbances and ROS production (Sharma et al. 2009). Indeed, there is no direct relationship between ND5 protein (encoded by the mitochondrial genome) and the mtDNA-CN because the expression machinery in mitochondria may be impaired which may result in decreased ND5 protein level despite the increased mtDNA-CN. This suggestion could be supported by the downregulation of the placental expression of POLG at the mRNA level however these results cannot be explained without the assessment of the protein level and activity of POLG which is one limitation of the present study. Also, this study showed a significant decline in citrate synthase (CS) activity, CS is a rate-limiting enzyme in the citrate cycle and is capable of catalyzing oxaloacetate and acetyl-CoA to citrate and its activity is considered a biomarker of mitochondrial content and function (Ortenblad et al. 2005).

In fetal muscle, the declined mtDNA-CN is associated with a reduction of the PGC-1α, NRF1, and Tfam proteins while at mRNA level, unexpectedly, NRF1 showed marked induction. This discrepancy in NRF1 expression at mRNA and protein suggests the existence of other post-transcriptional, translational, and/or post-translational regulatory pathways that need further investigation. It appears that the main determinant of mtDNA-CN in muscle is the protein levels of NRF1 and Tfam as indicated by the strong positive correlations between them. Under normal physiological conditions, NRF-1 is widely expressed, and the highest expression has been recognized in skeletal muscles (Roy and Tamuli 2009). Alone, NRF-1 was not enough to induce mitochondrial biogenesis, Baar et al., reported that the increased NRF-1 was not sufficient to initiate the expression of the proteins required for the assembly of functional mitochondria (Baar et al. 2003). In line with the present work, Kang et al. found a direct relation between Tfam and mtDNA, as heterozygous mutations of Tfam negatively affect mtDNA-CN, whereas homozygous mutation is embryonically lethal. Tfam heterozygous cells produce more inflammatory cytokines due to mitochondrial stress signaling. Hence, downregulation of Tfam expression resulted in mtDNA stress signaling. Levels of Tfam directly control mtDNA content, thus, any post-translational modifications that disrupt either the turnover or stability of Tfam could substantially impact the regulation of mtDNA abundance (Pejznochova et al. 2010; Kang et al. 2018).

The fetal muscles of fetuses of exposed mothers have higher ND5 protein content and POLG mRNA expression despite the declined mtDNA-CN which may be considered a compensatory mechanism to reserve the mitochondrial functions which showed marked decline as indicated by lower CS activity. Some investigators have found CS activity to decline with age in skeletal muscles which is significantly correlated with higher levels of mitochondrial oxidative damage (Figueiredo et al. 2009). These abnormalities in the fetal muscles are coupled with significant shifting in the redox status into oxidizing environments (as indicated by higher levels of GSSG and diminished GSH and GSH/GSSG ratio) that results in induced oxidative stress that manifested as increased lipid peroxidation (high MDA content) which induce inflammatory responses as indicated by marked elevation of the NF-κB at both mRNA and protein levels that may contribute to a decline in muscle mass, strength and functions. Therefore, prenatal mitochondrial dysfunction and decreased mitochondrial biogenesis in skeletal muscles may manifest later in life, causing long-term consequences on energy homeostasis (Zou et al. 2017).

The maintenance of muscle mass and its ability to function relies on a bioenergetic efficient mitochondrial network. This network is highly impacted by fusion and fission events. They have recently shown that the acute deletion of the fusion protein OPA1 induces muscle atrophy, systemic inflammatory response, precocious epithelial senescence, and premature death (Romanello et al. 2019).

Mitochondrial fission and fusion are essential processes in the maintenance of skeletal muscle function. Touvier et al., revealed that mitochondrial network dynamics influence muscle development and repair in a transgenic mouse line that overexpresses the fission-inducing protein DRP1 specifically in skeletal muscle. These mice displayed a drastic impairment in postnatal muscle growth, with a reorganization of the mitochondrial network and reduction of mtDNA quantity. Importantly the DRP1 overexpression activates the stress-induced pathway thus leading to attenuated protein synthesis and downregulation of the growth hormone pathway affecting muscle growth (Touvier et al. 2015).

In the fetal liver, the pre-conception exposure of young females to Sofosbovir contributed to mitochondrial dysfunction as indicated by a significant decline in the content of ND5 and CS activity. Although the expression of hepatic PGC-1α and Tfam has been downregulated at both mRNA and protein levels, the mtDNA-CN has not significantly changed which may indicate the redundant control of the mitochondrial homeostasis in the hepatic tissues due to the critical importance of mitochondria in the regulation of carbohydrates, lipid, and amino acid metabolism, energy production and the urea cycle, as well as regulating the innate immune response to control (Mansouri et al. 2018). Also, Sofosbuvir exposure causes fetal liver inflammation and increased oxidative stress which could contribute to the development and progression of liver diseases. In addition to mitochondrial fusion defects in the liver which may cause a non-alcoholic steatohepatitis-like phenotype and liver cancer. DRP1-mediated mitochondrial fragmentation observed in non-alcoholic fatty liver disease has been proposed to be a maladaptive process that exacerbates hepatic insulin resistance, steatohepatitis and cell death. Accordingly, both deletion and inhibition of hepatocyte-DRP1 activity in a preventative manner protected mice from high-fat diet-induced hepatic steatosis, insulin resistance and even body weight gain (Steffen et al. 2022).

These findings provide preliminary evidence for the possible prenatal mitochondrial consequences of pre-conception maternal exposure to Sofosbuvir that may have long-term adverse health impacting effects on the offspring. The mechanism(s) of pre-conception Sofosbuvir exposure-induced changes in the pregnancy outcomes and fetal and placental tissues is (are) unclear and needs intensive study. However, we can suggest a different possible mechanism that may participate: (1) Sofosbuvir may directly or indirectly affect the quality of the ova by disrupting the mitochondrial regulatory machinery that drives fetal abnormalities developed later during and after pregnancy, (2) Sofosbuvir may induce placental abnormalities and oxidative stress early in pregnancy that may affect the nutrient supply to the fetus which results in an increased risk of mtDNA mutations, leading to mitochondrial dysfunction. The extent and the interplay of these possible mechanisms need further investigations. The present findings require further proof of evidence by conducting future investigations that cover the limitations of the present study which include, the analysis of epigenetic, post-transcriptional, and translational mechanisms.

## Conclusion

From the present study, we can conclude that the preconception exposure of young female rats to sofosbuvir may induce changes in the maternal mitochondria before the induction of pregnancy that affects pregnancy outcomes, decrease fertility, and impact the mitochondrial biogenesis, dynamics and functions in the prenatal fetal liver, skeletal muscle and placental tissues by changing the pattern of fetal and placental expression of the genes controlling mitochondrial homeostasis and functions. Studies should be carried out to determine if these alterations are permanent or reversible. In the case that they are reversible, it is necessary to study the time necessary from the interruption of the treatment with Sofosbuvir and the pregnancy.

## Data Availability

The data and materials are available upon request.

## Abbreviations

HCV: Hepatitis C virus
mtDNA-CN: Mitochondrial DNA-copy number
NCCVH: National Committee for Control of Viral Hepatitis
DAA: Direct-acting antiviral
POLG: DNA polymerase γ
ROS: Reactive oxygen species
ETC: Electron transport chain
PGC-1α: Peroxisome proliferator-activated receptor-gamma coactivator-1 alpha
NRF-1: Nuclear transcription factors including nuclear respiratory factor-1
Tfam: Mitochondrial transcription factor A
GD 17: Gestational day 17
PBS: Phosphate buffer saline
MDA: Malondialdehyde
ND5: NADH dehydrogenase subunit-5
GSH: Glutathione
GSSG: Glutathione disulfide
DTNB: 5,5`-Dithiobis-(2-nitrobenzoic acid)
TNB: 5-Thio-2-nitrobenzoic acid
TBARS: Thiobarbituric acid reactive substances
TBA: Thiobarbituric acid
CRL: Crown-rump length
NF-κB: Nuclear factor-kappa B
CS: Citrate synthase
DRP1: Dynamin-related protein 1
Mfn: Mitofusin
OPA1: Optic atrophy protein 1

## References

[CR1] Abbade J, Klemetti MM, Farrell A, Ermini L, Gillmore T, Sallais J, Tagliaferro A, Post M, Caniggia I (2020). Increased placental mitochondrial fusion in gestational diabetes mellitus: an adaptive mechanism to optimize feto-placental metabolic homeostasis?. BMJ Open Diabetes Res Care.

[CR2] Abdeen AM, Essawy T, Mohammed SS (2019). Effect of sofosbuvir administration and its withdrawal on the submandibular salivary gland of adult male albino rats: a histological and ultra-structural study. Open Access Maced J Med Sci.

[CR3] Baar K (2003). Skeletal muscle overexpression of nuclear respiratory factor 1 increases glucose transport capacity. FASEB J.

[CR4] Chiaratti MR (2018). Oocyte mitochondria: role on fertility and disease transmission. Anim Reprod.

[CR5] Delany A, McCarthy F, Walsh S, Kenny L (2013). PP053. The role of peroxisome proliferator-activated receptor gamma co-activator 1-alpha in pregnancy. Pregnancy Hypertens.

[CR6] Dore GJ, Bajis S (2021). Hepatitis C virus elimination: laying the foundation for achieving 2030 targets. Nat Rev Gastroenterol Hepatol.

[CR7] Draper HH, Hadley M (1990). Malondialdehyde determination as index of lipid peroxidation. Methods Enzymol.

[CR8] El-Bassiouni EA, Helmy MH, Abou Rawash N, El-Zoghby SM, Kamel MA, Abou Rayah AN (2005). Embryopathy in experimental diabetic gestation: assessment of oxidative stress and antioxidant defence. Br J Biomed Sci.

[CR9] Figueiredo PA, Powers SK, Ferreira RM, Appell HJ, Duarte JA (2009). Aging impairs skeletal muscle mitochondrial bioenergetic function. J Gerontol Series A Biomed Sci Med Sci.

[CR10] Goldstein JA, Gallagher K, Beck C, Kumar R, Gernand AD (2020). Maternal-fetal inflammation in the placenta and the developmental origins of health and /disease. Front Immunol.

[CR11] Gomaa A, Allam N, Elsharkawy A, El Kassas M, Waked I (2017). Hepatitis C infection in Egypt: prevalence, impact and management strategies. HMER.

[CR12] Gowayed M (2020). Enhanced mitochondrial biogenesis is involved in the ameliorative action of creatine supplementation in rat’s soleus and cardiac muscles. Exp Ther Med.

[CR13] Griffith OW (1980). Determination of glutathione and glutathione disulfide using glutathione reductase and 2-vinylpyridine. Anal Biochem.

[CR14] Hagen S (2002). SPSS in practice. Nurse Res.

[CR15] Herrera E, Desoye G (2016). Maternal and fetal lipid metabolism under normal and gestational diabetic conditions. Horm Mol Biol Clin Invest.

[CR16] Holland O (2017). Review: placental mitochondrial function and structure in gestational disorders. Placenta.

[CR17] Issa NM, El-sherief NM (2017). Histological and immunohistochemical studies on the cornea and retina of sofosbuvir treated rats. Austin J Anat.

[CR18] Kamel MA (2012). Prenatal effects of different intra-uterine milieus on fetal glucose sensing mechanisms during gestation in rats. Journal of Diabetes & Metabolism.

[CR19] Kang I, Chu CT, Kaufman BA (2018). The mitochondrial transcription factor TFAM in neurodegeneration: emerging evidence and mechanisms. FEBS Lett.

[CR20] Kim SJ (2017). Ginsenoside Rg3 restores hepatitis C virus–induced aberrant mitochondrial dynamics and inhibits virus propagation. Hepatology.

[CR21] Lowry OH, Rosebrough NJ, Farr AL, Randall RJ (1951). Protein measurement with the Folin phenol reagent. J Biol Chem.

[CR22] Ma K, Chen G, Li W, Kepp O, Zhu Y, Chen Q (2020). Mitophagy, mitochondrial homeostasis, and cell fate. Front Cell Dev Biol.

[CR23] Mandò C (2014). Placental mitochondrial content and function in intrauterine growth restriction and preeclampsia. Am J Physiol Endocrinol Metab.

[CR24] Mansouri A, Gattolliat C-H, Asselah T (2018). Mitochondrial dysfunction and signaling in chronic liver diseases. Gastroenterology.

[CR25] May-Panloup P (2016). Ovarian ageing: the role of mitochondria in oocytes and follicles. Hum Reprod Update.

[CR26] Omran D (2018). Towards hepatitis C virus elimination: Egyptian experience, achievements and limitations. World J Gastroenterol.

[CR27] Ortenblad N, Mogensen M, Petersen I, Hojlund K, Levin K, Sahlin K (2005). Reduced insu-lin-mediated citrate synthase activity in cultured skeletal musclecells from patients with type 2 dia-betes: evidence for an intrinsic oxidativeenzyme defect. Biochim Biophys Acta.

[CR28] Pejznochova M (2010). Mitochondrial DNA content and expression of genes involved in mtDNA transcription, regulation and maintenance during human fetal development. Mitochondrion.

[CR29] Popov L-D (2020). Mitochondrial biogenesis: an update. J Cell Mol Med.

[CR30] Romanello V, Scalabrin M, Albiero M, Blaauw B, Scorrano L, Sandri M (2019). Inhibition of the fission machinery mitigates OPA1 impairment in adult skeletal muscles. Cells.

[CR31] Roy D, Tamuli R (2009). NRF1 (nuclear respiratory factor 1). Atlas Genet Cytogenet Oncol Haematol.

[CR32] Saad MI (2016). Maternal obesity and malnourishment exacerbate perinatal oxidative stress resulting in diabetogenic programming in F1 offspring. J Endocrinol Invest.

[CR33] Sharma L, Lu J, Bai Y (2009). Mitochondrial respiratory complex i: structure, function and implication in human diseases. Curr Med Chem.

[CR34] Shepherd D, Garland PB (1969). Citrate synthase from rat liver. Methods Enzymol.

[CR35] Spearman CW, Dusheiko GM, Hellard M, Sonderup MH, C.  (2019). The Lancet.

[CR36] Spera AM, Eldin TK, Tosone G, Orlando R (2016). Antiviral therapy for hepatitis C: Has anything changed for pregnant/lactating women?. World J Hepatol.

[CR37] Steffen J, Ngo J, Wang SP, Williams K, Kramer HF, Ho G, Rodriguez C, Yekkala K, Amuzie C, Bialecki R, Norquay L, Nawrocki AR, Erion M, Pocai A, Shirihai OS, Liesa M (2022). The mitochondrial fission protein DRP1 in liver is required to mitigate NASH and prevents the activation of the mitochondrial ISR. Mol Metab.

[CR38] Stoker ML, Newport E, Hulit JC, West AP, Morten KJ (2019). Impact of pharmacological agents on mitochondrial function: a growing opportunity?. Biochem Soc Trans.

[CR39] Touvier T, De Palma C, Rigamonti E, Scagliola A, Incerti E, Mazelin L, Thomas JL, D'Antonio M, Politi L, Schaeffer L, Clementi E, Brunelli S (2015). Muscle-specific DRP1 overexpression impairs skeletal muscle growth via translational attenuation. Cell Death Dis.

[CR40] Viengchareun S (2007). Mitochondrial toxicity of indinavir, stavudine and zidovudine involves multiple cellular targets in white and brown adipocytes. Antivir Ther.

[CR41] Vishnyakova PA (2016). Mitochondrial role in adaptive response to stress conditions in preeclampsia. Sci Rep.

[CR42] Waked I, Esmat G, Elsharkawy A, El-Serafy M, Abdel-Razek W, Ghalab R, Elshishiney G, Salah A, Abdel Megid S, Kabil K, El-Sayed MH (2020). Screening and treatment program to eliminate hepatitis C in Egypt. N Engl J Med.

[CR43] Zou TD (2017). Mitochondrial biogenesis is decreased in skeletal muscle of pig fetuses exposed to maternal high-energy diets. Animal.

